# Transforming patient recruitment in clinical trials: Communication training to manage uncertainties about risks and benefits

**DOI:** 10.1017/cts.2026.10725

**Published:** 2026-03-04

**Authors:** Ekaterina Malova, Xiaofeng Jia, Rutendo E. Chimbaru, Tyler R. Harrison, Kallia O. Wright, Hanna Birenbaum Cooper, Susan E. Morgan

**Affiliations:** 1 University of Rochester William E Simon Graduate School of Business, USA; 2 Communication, Bowling Green State University, USA; 3 Communication Studies, University of Miami - Coral Gables Campus, USA

**Keywords:** Clinical trial recruitment, uncertainty management theory, informed consent, communication training

## Abstract

**Purpose:**

Effective communication of risks and benefits is essential to ethical clinical trial recruitment, yet clinical research coordinators (CRCs) often lack formal training in how to navigate participant uncertainty. This study examined whether targeted communication training could enhance CRCs’ ability to disclose risks and benefits clearly and ethically.

**Approach or Design:**

A mixed-methods, pre–post design compared CRCs who received communication training with those in a control group.

**Setting:**

Standardized patient (SP) interactions simulating clinical trial recruitment conversations.

**Participants:**

Twenty-four CRCs participated across intervention and control groups.

**Intervention:**

A communication training program grounded, focusing on transparent disclosure, benefit framing, and participant engagement strategies.

**Method:**

Qualitative content analysis of SP interaction transcripts guided by uncertainty management theory examined risk disclosure, mitigation strategies, benefit framing, and engagement. Frequency analysis tracked changes in the number and specificity of risk/benefit mentions over time.

**Results:**

CRCs in the intervention group demonstrated more structured and transparent risk disclosure, greater use of participant-centered benefit framing, and improved interpersonal engagement. Frequency analysis showed significant increases in both the number and specificity of risk and benefit mentions post-training. Control group CRCs showed minimal change.

**Conclusion:**

Targeted communication training enhances CRCs’ ability to manage participant uncertainty, improving both ethical standards and informed consent quality in clinical trial recruitment.

## Introduction

Effective risk communication with potential participants in clinical trials is crucial for informed decision making regarding clinical trial participation. Clinical trials are widely regarded as the benchmark for evaluating new clinical treatments and are therefore used as the foundation for developing clinical guidelines and advancing existing therapies [1]. Yet, previous research reported that 25% of cancer trials failed to enroll enough participants, with 18% of clinical trials concluding with less than half of their target enrollment after three or more years. This suggests that many promising interventions for various illnesses remain untested [2–4]. Thus, unsuccessful recruitment is a major factor contributing to the failure of clinical trials [5,6]. Factors contributing to low rates of accrual are related to both potential participants as well as communication by medical staff and recruitment professionals. Previous studies have reported that patients’ concerns about participating in clinical trials include potential unknown side effects from the study treatment [7], the length of the study [8], and being assigned to a control group without an active study drug [9]. Additionally, difficulties in communication between medical professionals and patients further contribute to low accrual rates [10,11].

A few training programs have been specifically designed to meet the educational needs of clinical research personnel. A systematic review identified only 22 published articles describing such training initiatives [12]. The reported outcomes largely focused on participants’ self-confidence, satisfaction, and understanding of key aspects of clinical trials [12–14] While some programs noted improvements in communication skills, few provided details about the specific skills gained [15–19]. Improved risk communication strategies are needed to balance the benefits and risks of clinical trials effectively [20,21]. There is no clear consensus on optimal methods for enhancing participant comprehension or willingness to participate [22], which leads to clinical research coordinators (CRCs) and researchers facing challenges in identifying, defining, communicating, and managing risks [23]. To address patient concerns and uncertainty regarding clinical trial participation, communication focused training programs are needed for professionals who recruit for clinical trials. The purpose of this study is to assess whether structured training for CRCs leads to improvements in uncertainty management strategies when communicating risks and benefits of clinical trial participation, fostering a more open and participant-friendly clinical trial recruitment process.

## Uncertainty management in clinical trial recruitment

Uncertainty management theory (UMT) [24] provides a robust conceptual framework for understanding how individuals experience, interpret, and respond to uncertainty in health contexts [25,26]. Unlike theories that assume uncertainty is inherently aversive and must be reduced, UMT posits that individuals manage uncertainty in diverse ways – seeking to reduce, maintain, or even increase it – depending on how they appraise its meaning and implications [24,27]. In clinical research settings, where patients face complex decisions about trial participation, uncertainty is particularly salient. Patients may feel unsure about trial protocols, risks, personal benefits, or their own eligibility, all of which carry cognitive and emotional weight. Thus, the role of the CRC is not simply to transfer information, but to engage in communication that helps patients manage this uncertainty in ways that support informed decisions. Based on UMT, individuals experiencing uncertainty may display a wide range of emotional responses, including negative, positive, neutral, or mixed [28]. Negative emotions are typically associated with uncertainty perceived as threatening, which can hinder decision-making. For instance, being assigned to a control group in a clinical trial for a chronic illness may elicit fear or disappointment. In contrast, positive emotions may arise when uncertainty brings hope or optimism, such as when enrolling in a clinical trial for a terminal illness offers a chance at a novel treatment. In cases of mixed emotional responses, uncertainty can be appraised as both threatening and promising. For example, a person with a terminal illness may consider trial participation as a hopeful opportunity while remaining concerned about potential side effects [29]. UMT has been applied across a variety of health communication contexts, with researchers examining its relevance to populations affected by HIV/AIDS, genetic counseling sessions, and how patients seek and process medical information [27,30–32]. However, there remains a notable gap in applying communication-based frameworks to understand how CRCs manage participant uncertainty during trial recruitment. Most existing work has focused on interventions that offer emotional, informational, or social support to participants, such as training programs that improve coping or provide clearer messaging [25,33–35].

By applying UMT to this context, we aim to move beyond a compliance-based view of informed consent and toward a more relational understanding of how CRCs can ethically and empathetically guide patients through the uncertainty inherent in clinical research participation. To guide this investigation, we pose the following research questions:

1. How do CRCs communicate risks to manage uncertainty about clinical trial participation before and after the training?
2. How do CRCs frame participant benefits to manage uncertainty about clinical trial enrolment before and after the training?
3. Does the use of participant-centered strategies to manage uncertainty improve after training?

## Methods

### Participants and procedures

The training program was held at a university-affiliated medical center in a flexible learning space that supported interactive activities such as role-plays and group work. Participants (*n* = 24), who were research coordinators and physicians involved in clinical trial recruitment, were recruited from across the institution and compensated upon completing all study components. Detailed demographic information for intervention and control group participants is presented in Tables 1 and 2. The intervention group completed video-recorded simulated recruitment sessions with standardized patients (SPs) before and after the training, while the control group participated in two sessions prior to receiving the delayed intervention. Recruitment simulations took place in a mock clinical setting designed to replicate a typical exam room. SPs were instructed to express skepticism about trial participation, including concerns about being treated like a “guinea pig,” and to disclose a family history of heart disease to evoke empathetic responses. The dataset comprises transcripts of video-recorded SP simulations conducted at two time points: Time 1 (Pre-Training), when CRCs presented study information prior to training, and Time 2 (Post-Training), for the intervention group after completing the communication training. These data were compared to control group transcripts, as control participants received the training only after the Time 2 assessment.

**Table 1.** Intervention group demographics

Demographic category	n (%) of participants (*N* = 15)
Race	
American Indian or Alaska Native	0 (0%)
Asian or Pacific Islander	1 (6.7%)
Black or African American	2 (13.3%)
Middle Eastern	1 (6.7%)
White or Caucasian	11(73.3%)
Prefer not to say	0 (0%)
Ethnicity	
Hispanic/Latino	9 (60%)
Non-Hispanic/Latino	6 (40%)
Age	25–43 *M* = 33.40, *SD* = 4.72
Gender	
Female	11 (73.3%)
Male	4 (26.7%)
Education	
Bachelor’s degree	2 (13.3%)
Master’s degree (MA, MPH)	8 (53.3%)
Doctorate degree	0 (0%)
Medical doctor	5 (33.3%)

**Table 2.** Control group demographics

Demographic category	*n* (%) of participants (*N* = 12)
Race	
American Indian or Alaska Native	0 (0%)
Asian or Pacific Islander	0 (0%)
Black or African American	1 (8.3%)
Middle Eastern	0 (0%)
White or Caucasian	10 (83.3%)
Prefer not to say	1 (8.3%)
Ethnicity	
Hispanic/Latino	8 (66.7%)
Non-Hispanic/Latino	4 (33.3%)
Age	27–57 *M* = 37.92, *SD* = 11.15
Gender	
Female	9 (27%)
Male	3 (25%)
Education	
Bachelor’s degree	6 (50%)
Master’s degree (MA, MPH)	2 (16.7%)
Doctorate degree	1 (8.3%)
Medical doctor	3 (25%)

### Clinical trial training program

The training program was developed after formative research [35–37] and focused on communication practices addressing concerns of marginalized populations about clinical trial participation. The 4.5-hour session combined interactive lectures, exercises, role-plays, and group discussions to strengthen CRCs’ skills. It began with demonstrations of ineffective and effective recruitment conversations, followed by group discussion. Instruction covered verbal and nonverbal strategies, empathetic engagement, and cultural considerations. Activities included active listening, translating consent language into lay terms, and small-group role-plays simulating recruitment. The session concluded with a debrief to reflect on lessons learned and integrate takeaways. The control group (*n* = 12) completed SP simulations at both time points but did not receive training until after the second interaction. This design supported both within-subject and between-group comparisons.

### Analysis

To assess changes in CRC communication about study risks and benefits, we first conducted a frequency analysis of transcript codes from mock recruitment sessions. We counted the number of times CRCs referenced risks and benefits at two points: before training (Time 1) and after training (Time 2). This quantitative content analysis tracked shifts in the prevalence and emphasis of disclosures, allowing us to examine whether training influenced how CRCs balanced risks and benefits.

We then applied qualitative content analysis [39] to explore shifts in risk communication, benefit framing, and participant engagement. A structured coding framework focused on three categories: (1) risk disclosure and mitigation strategies (naming risks and explaining monitoring procedures), (2) benefit framing (direct, potential, societal), and (3) participant engagement (open dialogue). Coding was iterative and collaborative: transcripts were read holistically, open coded, organized into themes, and checked for inter-coder reliability through independent coding and team discussion. To isolate training effects from protocol familiarity, we conducted the same analysis on control group transcripts, applying the identical coding framework to their pre- and post-intervention sessions.

## Results

### The frequency of risks and benefits disclosure

**Figure 1.**
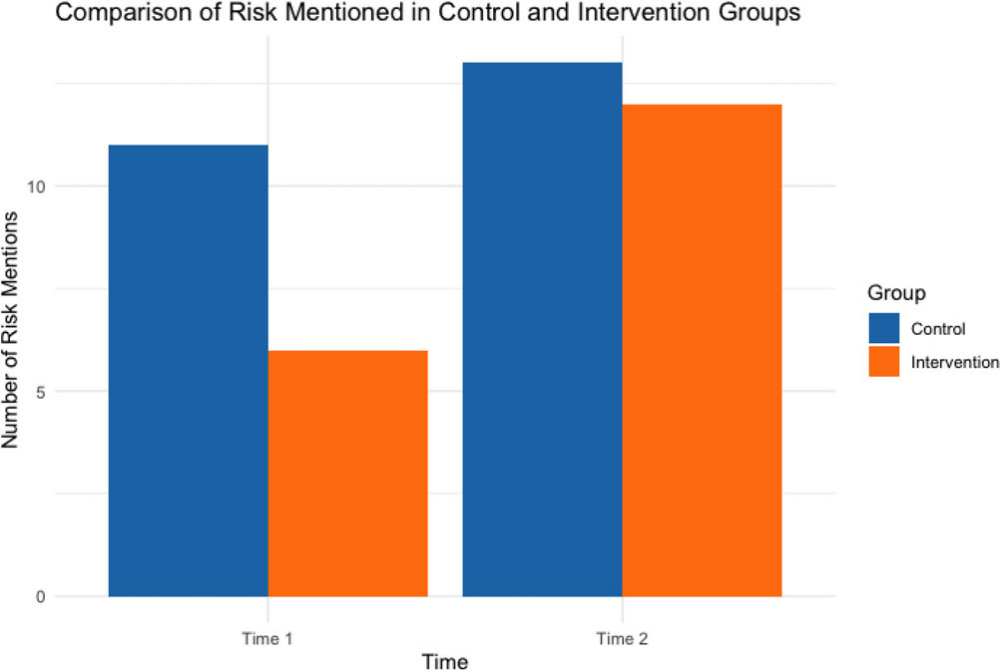
The comparison of risks mentions in control and intervention groups.

To answer RQ1 and RQ2 quantitively, the frequency analysis was performed. The descriptive statistics for the number of risks mentioned by CRCs before and after the interventions are presented in Table 3 and Figure 1. In the control group, the number of risk mentioned increased slightly from Time 1 (*M* = 0.92, SD = 0.90) to Time 2 (*M* = 1.08, SD = 1.04). In the intervention group, the number of risks mentioned significantly increased from Time 1 (*M* = 0.50, SD = 0.76) to Time 2 (*M* = 1.00, SD = 0.77). These results suggest that the intervention has been effective in encouraging CRCs to discuss risks more explicitly with patients.

**Table 3.** The comparison of the number of risks mentions

Control group			Intervention group		
ID	Time 1 risks	Time 2 risks	ID	Time 1 risks	Time 2 risks
1	0	0	2	0	1
5	0	2	3	0	1
7	2	2	6	1	1
10	2	0	8	0	1
12	1	1	9	0	2
13	0	1	11	1	2
18	2	3	15	1	0
19	1	1	16	0	1
21	0	0	17	1	0
23	2	2	22	1	1
24	1	0	25	1	1
27	0	1	26	0	1
Total	11	13	Total	6	12

* ID 4, 14, and 20 were excluded from the comparison due to the missing data.

**Figure 2.**
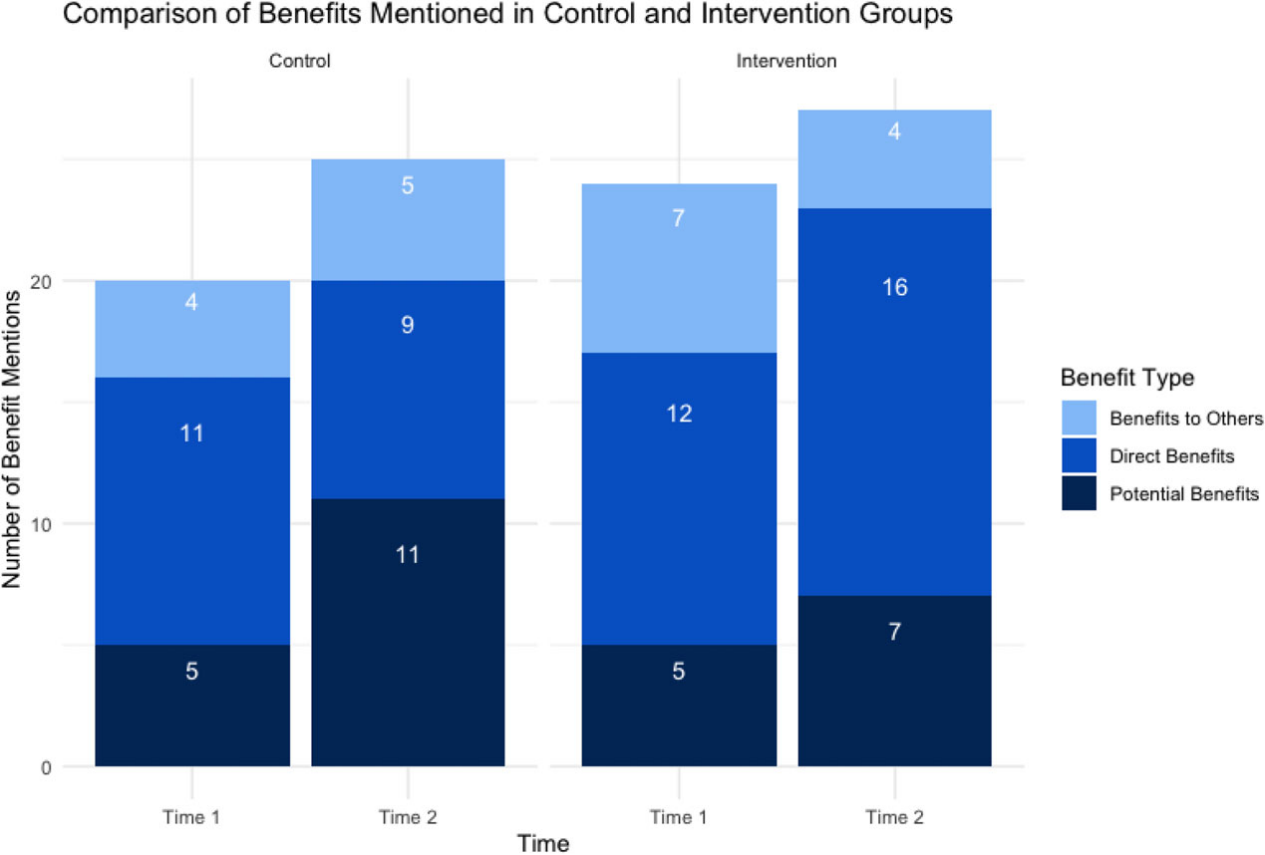
The comparison of different types of benefits mentioned in control and intervention groups.

The comparison of benefits mentioned by CRCs in the control and intervention groups before and after the intervention is illustrated in Table 4 and Figure 2. The results indicate distinct patterns in how CRCs frame participant benefits over time. In the control group, the total number of benefits mentioned increased from Time 1 (*n* = 21) to Time 2 (*n* = 25). Specifically, mentions of potential benefits rose from 5 to 11, while direct benefits decreased from 12 to 9. Mentions of benefits to others remained relatively stable, increasing from 4 to 5. In the intervention group, the total number of benefits mentioned also increased from 24 at Time 1 to 27 at Time 2. Mentions of direct benefits significantly increased from 12 to 16, while potential benefits mentioned also rose from 5 to 7. This shift suggests that the intervention may have influenced CRCs to place more emphasis on direct and potential benefits. Overall, the results imply that the intervention enhanced CRCs’ communication about direct and potential benefits, reflecting a more participant-centered approach.

**Table 4.** The comparison of the number of benefits (direct benefits, potential benefits, and benefits to others)

		Time 1				Time 2			
	ID	Direct benefits	Potential benefits	Benefits to others	Total benefits	Direct benefits	Potential benefits	Benefits to others	Total benefits
Control group	1	3	0	0	3	1	0	0	1
	5	0	0	0	0	0	2	0	2
	7	1	1	1	3	1	1	1	3
	10	3	0	0	3	1	0	1	2
	12	1	1	0	2	0	2	1	3
	13	0	0	1	1	2	2	1	5
	18	0	0	0	0	0	1	0	1
	19	2	0	0	2	2	0	0	2
	21	0	0	0	0	0	0	0	0
	23	1	1	1	3	1	3	1	5
	24	1	1	0	2	0	0	0	0
	27	0	1	1	2	1	0	0	1
	Total	12	5	4	21	9	11	5	25
Intervention group	2	2	0	1	3	3	0	0	3
	3	0	0	0	0	1	1	0	2
	6	2	0	1	3	0	1	0	1
	8	3	2	1	6	1	1	1	3
	9	0	1	0	1	3	0	0	3
	11	0	0	0	0	2	2	1	5
	15	0	0	1	1	1	0	0	1
	16	0	2	0	2	2	0	0	2
	17	1	0	1	2	1	1	1	3
	22	1	0	1	2	0	1	1	2
	25	3	0	0	3	1	0	0	1
	26	0	0	1	1	1	0	0	1
	Total	12	5	7	24	16	7	4	27

### Qualitative analysis of CRCs pre- and post-training communication strategies

The qualitative analysis revealed post-training improvements in CRCs’ risk disclosure, mitigation strategies, benefit framing, and participant engagement. Training enhanced transparency, participant-centered framing, and uncertainty management. Findings are presented across three domains – risk communication, benefits, and engagement – using within-subject comparisons (Time 1 vs. Time 2) and control group contrasts. Please see summary Tables 5 and 6.

**Table 5.** Intervention group: CRC comparison across three domains pre- and post-training

CRC ID	Risk communication	Benefit framing	Participant engagement
2	Time 1: General reassurance, lacked specific risks Time 2: Provided written risk documents, invited follow-up	Time 1: Focused on research contribution Time 2: Balanced personal benefits and cost savings	Time 1: Structured and formal Time 2: Conversational and inviting
6	Time 1: Compared risks to daily life, lacked specifics Time 2: Listed side effects, discussed safety monitoring	Time 1: Emphasized scientific goals Time 2: Explained personal monitoring and better outcomes	Time 1: Dense information, limited interaction Time 2: Checked for understanding, encouraged dialogue
9	Time 1: Technical, minimal specificity Time 2: Detailed side effects, emphasized monitoring	Time 1: Focused on trial design Time 2: Highlighted access to care, monitoring benefits	Time 1: Formal, abstract Time 2: Personalized, acknowledged participant needs
8	Time 1: “Minimal risk,” avoided detail Time 2: Explained dizziness/fatigue	Time 1: General long-term framing Time 2: Connected participation to improved care and oversight	Time 1: Fact-based, low interactivity Time 2: More open-ended, validated concerns
3	Time 1: Reassurance via FDA approval Time 2: Named side effects, described physician-led mitigation	Time 1: Focused on study process Time 2: Framed benefits in terms of health and compensation	Time 1: Defensive to skepticism Time 2: Invited follow-up, validated emotions
15	Time 1: Technically dense, unclear relevance Time 2: Simplified and clarified risk oversight and symptoms	Time 1: Emphasized research aim Time 2: Linked trial to stroke prevention, participant monitoring	Time 1: Formal, lecture-style Time 2: Open-ended, participant-guided tone
16	Time 1: Generic risk reassurance Time 2: Framed risks in relation to participant safety	Time 1: Trial-focused framing Time 2: Explained follow-ups and enhanced support	Time 1: Lightly personalized Time 2: Dialogue-oriented, emotionally attuned
17	Time 1: Procedural, avoided risks Time 2: Named side effects, connected testing to safety	Time 1: Emphasized long-term contribution Time 2: Personal benefit of follow-ups	Time 1: Transactional Time 2: Warm, collaborative, emotionally responsive
11	Time 1: Overly clinical, used medical jargon without mitigation strategies Time 2: Clearly explained risks and linked to safety measures	Time 1: General health improvement emphasis Time 2: Framed participation as an opportunity for closer oversight and proactive care	Time 1: Formal and information-heavy Time 2: Validated participant agency, encouraged shared decision-making
22	Time 1: Already clear and comprehensive Time 2: Maintained clarity, emphasized patient control	Time 1: Balanced and participant-centered Time 2: Slightly more personalized	Time 1: Highly engaging and empathetic Time 2: Continued strength and responsiveness
26	Time 1: Vague, unstructured, confusing Time 2: More empathetic tone, but unclear on side effects	Time 1: Claimed “no direct benefit” Time 2: Some improvement; linked to family risk and oversight	Time 1: Disorganized and scripted Time 2: Friendlier tone, better validation but still inconsistent
25	Time 1: Limited specificity, deferred to documentation Time 2: Provided clearer explanation of side effects and monitoring	Time 1: Framed benefits abstractly and passively Time 2: Connected participation to long-term health outcomes and proactive care	Time 1: Professional but impersonal Time 2: More conversational, expressed care, and encouraged reflection

**Table 6.** Control group: CRC comparison across three domains pre and post training

CRC ID	Risk communication	Benefit framing	Participant engagement
1	Time 1: General reassurance, vague Time 2: Similar patterns	Time 1: Slightly tailored benefit Time 2: Still impersonal	Time 1: Structured, passive Time 2: Minimal change
5	Time 1: Minimally mentioned risks Time 2: Slightly clearer, still vague	Time 1: Scientific purpose Time 2: Same framing, more assurance	Time 1: One-directional Time 2: Slightly warmer tone
7	Time 1: Risk omitted Time 2: Risks still downplayed	Time 1: Generalized benefit Time 2: No elaboration	Time 1: Limited engagement Time 2: Slightly more interactive
10	Time 1: Technical, unclear Time 2: Minor improvement	Time 1: Abstract framing Time 2: “Big team” concept added	Time 1: Scripted Time 2: Slightly animated, still limited dialogue
12	Time 1: Technical, vague Time 2: No change	Time 1: Procedural explanation Time 2: Repetition of same logic	Time 1: Formal Time 2: Unchanged
13	Time 1: No side effect detail Time 2: Reiterated logic, risks minimized	Time 1: Institutional framing Time 2: Unchanged	Time 1: Passive Time 2: Passive
18	Time 1: Risks unclear Time 2: Minor additions	Time 1: Generic future benefit Time 2: Added family risk framing	Time 1: Compassionate tone Time 2: More follow-up, slightly better flow
19	Time 1: FDA reassurance only Time 2: Added “abnormal results” reference	Time 1: Goal of prevention Time 2: No new framing	Time 1: Procedural Time 2: Slightly warmer
21	Time 1: Unclear, limited Time 2: Same safety language, vague	Time 1: No detail Time 2: Mentioned monitoring	Time 1: Detached Time 2: More accommodating
23	Time 1: Procedural, no risks Time 2: Slight oversight detail added	Time 1: Community-based framing Time 2: Reassurance added	Time 1: Passive Time 2: Slightly conversational
24	Time 1: Deferred to forms Time 2: Same summary style	Time 1: Scientific focus Time 2: No new benefit	Time 1: Formal Time 2: Slightly more validation
27	Time 1: Oversight mentioned Time 2: Added safety details	Time 1: Abstract Time 2: Added benefits	Time 1: Informative Time 2: More options, still formal

#### RQ1: Risk disclosure and mitigation strategies as a result of communication training

**Pre-Training (Time 1).** Before training, CRCs in the intervention group communicated about risks using general language, vague reassurances, and procedural explanations that lacked participant-centered clarity. For example, a CRC framed the study in terms of its routine nature but avoided risk specifics:

*We’re going to try a new scheme of medications and see how they work on you. That’s the reason why you are here talking to me. We’re going to try to use different levels of medications to try to control your blood pressure.* (CRC 3)

This language conveys the logic behind the study but lacks a clear discussion of what might go wrong or how participants would be protected. Other CRCs provided more technical information but used terminology that may not be easily understood by a lay participant: *“Side effects… could be electrolyte disturbance like raising your blood sodium or potassium… palpitations or maybe severe conditions like dyspnea”* (CRC 11). While medically accurate, this description was unstructured and overly clinical, potentially increasing uncertainty and increasing perceptions of risk. It also lacked connection to any monitoring or mitigation plan, leaving the participant unclear about how their health would be protected.

**Post-Training (Time 2).** Post-training, CRCs were significantly more transparent about risks, outlining specific side effects and safety protocols to address participant concerns. They also provided clearer mitigation strategies:

*I’m not sure if you noticed where we have this, the IRB, which is an institutional review board, and you could just think about them as the police for researchers. So, if we’re ever doing anything that’s not okay or in any way, shape, or form, puts you in risk or in danger, we’re 100% stopped by them.* (CRC 9)

Beyond listing side effects, CRCs explained how risks would be managed. A key improvement at Time 2 was integrating specific monitoring and response measures:

*So, there is this oversight that we have over studies that we do no harm and we keep a constant review of both groups to see if both groups are benefiting or not having any sort of response at all. And again, if one group seems to be benefiting significantly more than the other group, then we’re going to go ahead and reevaluate whether or not we push you into the [intervention] group.* (CRC 17)

Another change was framing risk in a participant-centered way. When the SP said, *“I don’t really like needles,”* the CRC responded with mitigation strategies:

*It could be, it’s scary, uncomfortable, but we will do our best so that you receive people that are experienced and not someone who doesn’t know how to draw blood. The doctor would be monitoring everything with us, so it’s not like you’re going to be dealing with any side effects on your own.* (CRC 6)

This shift shows how CRCs moved from vague reassurances to structured, participant-centered explanations that enhanced understanding and trust. Post-training communication emphasized side effects, monitoring protocols, and patient-centered reassurances.

In contrast, control group CRCs showed minimal change in risk communication from Time 1 to Time 2. Initially, they relied on vague reassurances (e.g., citing FDA approval or labeling the study “minimal risk”) without addressing side effects or safety monitoring. This pattern persisted: at Time 2, CRCs often repeated that the medications were “already approved” or deferred side effect information to written materials. A few added brief mentions of side effects or oversight committees, but these remained superficial. Overall, risk communication was general, procedural, and abstract, with little evidence of uncertainty management.

#### RQ2: Benefit framing by CRCs as a function of training

**Pre-Training (Time 1).** Before training, CRCs frequently framed study benefits in terms of broader scientific value or potential long-term outcomes, often neglecting to address immediate and tangible benefits for participants. In many cases, this approach left participants uncertain about the personal relevance or advantages of joining the study. Several CRCs emphasized future societal gains without explaining how participants might directly benefit. For instance, CRC 3 stated, “*What we’re getting is all the data. So what we’re going to use is the results of your blood pressure, of your lab results.”* CRC 17 noted that, *“It might not benefit you at the moment, but it will be a huge data for us to benefit someone else in the future.”* Additionally, CRC 26 stated bluntly, *“There are going to be no direct benefits to you.”* Statements like these reinforce the altruistic or scientific purpose of the study but do little to manage participant uncertainty or foster engagement. As a result, SPs often responded with skepticism or disappointment, particularly when it became unclear whether they would receive individualized attention or improved care during the trial.

**Post-Training (Time 2).** Following the training, most CRCs made substantial improvements in how they framed benefits, aligning their messaging with participant-centered care and individualized health outcomes. Rather than leading with abstract or collective gains, CRCs began to clearly articulate how participants might benefit from enhanced medical monitoring, better blood pressure control, or improved access to care. For example, several CRCs emphasized that frequent follow-ups and medical oversight were a built-in benefit of trial participation: *“Being part of this study means you actually get more tailored care than you would in standard treatment”* (CRC 16). They would also highlight the benefits of being in the study, despite it being a randomized trial.

*Even if you’re randomized to the same group, like receiving the same medication, the good thing is that you will be having a follow-up every month for the first three months and then after the three months, every three months.* (CRC 17)

This reframing of routine procedures as participant benefits helps manage uncertainty and increase motivation for enrollment. CRCs also began linking participation to long-term health improvements, such as preventing cardiovascular events or refining treatment plans:

*If we feel like, you know, something else might be better for you, we might bring in more often. We’ll take, you know, samples of your blood, we’ll be checking your overall health, because as you know, with hypertension, it can lead to stroke. It can lead to a heart attack.* (CRC 22)

Some CRCs, like CRC 22, offered empathetic comments by drawing on their personal experiences*: “I understand. My dad has a history of high blood pressure, so it’s always something that I’m mindful of as well.”* These improvements reflect a shift from neutral or impersonal scientific discourse to relational and trust-building communication, which better aligns with ethical standards in informed consent and uncertainty management.

As with risk communication, control group CRCs showed little change in how they framed benefits. At Time 1, benefits were described mainly in abstract or population-level terms (e.g., contributing to science, improving treatments for others), and this persisted at Time 2. Few mentioned concrete personal benefits (such as blood pressure control or frequent care), and those references were brief and untailored. Compared to the intervention group, control CRCs rarely linked participation to individualized care, close monitoring, or health improvements, and generally failed to present procedures as participant-centered benefits.

#### RQ3: Participant engagement as a result of communication training

**Pre-Training (Time 1).** Prior to training, many CRCs adopted a unidirectional style of communication, often defaulting to scripted delivery of study information without tailoring their approach to participants’ concerns or emotional cues. Engagement was typically limited to asking if the participant had questions at the end of the interaction, rather than actively encouraging dialogue or checking comprehension throughout. For instance, in several Time 1 interactions, CRCs delivered long blocks of information and concluded with perfunctory prompts: *“Let me know if you have any questions.” or “Is that okay with you?.”* These statements, while offering an opening, placed the burden on the participant to initiate further conversation rather than fostering an atmosphere of collaboration. Additionally, some CRCs missed opportunities to validate participant concerns or invite deeper conversation. For example, when participants raised questions about racial disparities in research or risks of being treated as a “guinea pig,” CRCs often responded with defensiveness or diverted the conversation without explicitly acknowledging participant emotion: “*We’re looking for patients from everywhere”* or *“This is standard research. It’s not like that.”* These types of responses, while factually accurate, may have inadvertently shut down further dialogue by failing to proceed with questions about the participant’s perspective.

**Post-Training (Time 2).** After training, CRCs adopted a more conversational and participant-centered communication style, leading to significantly improved engagement across most transcripts. CRCs began using open-ended questions and actively encouraged dialogue, such as: *“What are your thoughts so far?”* or *“Do you have any questions about what I just explained?.”* These approaches not only validated participant voices but also fostered a two-way exchange of information. In contrast to the “ask-at-the-end” approach seen in Time 1, CRCs in Time 2 were more likely to pause throughout the conversation and check for comprehension. Also, when participants shared personal experiences or hesitations, CRCs responded with greater emotional resonance: *“I’m very sorry to hear about your parents. I had a similar something that happened to me recently. I lost an auntie because of a condition that she had” (CRC 17).*

These moments reflected an increase in affective attunement, which is essential for building trust.

Post-training, most CRCs showed improved affective engagement, conversational fluency, and validation of concerns, marking a shift toward relational communication. These changes align with uncertainty management goals, supporting ethical enrollment and informed decision-making. In contrast, control group CRCs displayed minimal change. They maintained a scripted, professional tone at both time points, with Time 2 transcripts still reflecting a uni-directional style. CRCs typically asked for questions only at the end and rarely checked comprehension; emotional validation and open-ended questions were uncommon.

Overall, control group CRCs did not substantially improve in risk, benefit, or engagement strategies. The contrast with the intervention group indicates these gains were training-dependent rather than naturally occurring.

## Discussion

The findings of this study demonstrate that CRCs who received structured communication training exhibited significant improvements in risk disclosure, benefit framing, and participant engagement strategies. Using UMT [24] as a guiding framework, we interpret these shifts as evidence that structured, transparent, and participant-centered communication contributes to more effective engagement with potential clinical trial participants. These improvements align with UMT by showing that training equips CRCs with skills to address participant uncertainty more effectively. The following discussion explores how these findings advance understanding of CRC communication and clinical trial recruitment.

### Managing uncertainty through risk disclosure and mitigation

Before training, CRCs often gave vague or minimized descriptions of risks, leaving participants uncertain about side effects or safety measures. UMT holds that individuals evaluate uncertainty not only cognitively but also in terms of emotional and relational significance. Ambiguous or withheld information is appraised as threatening, leading to anxiety or mistrust [24,27]. Time 1 findings reflect this: SP frequently requested clarification, indicating that initial messages failed to support decision-making.

Post-training, CRCs proactively addressed risks, offering specific details and clear mitigation strategies. UMT emphasizes that communication helps individuals appraise and manage uncertainty rather than eliminate it [24]. Transparent disclosure supports participants in interpreting uncertainty and regaining control. Prior studies confirm that participants want detailed risk information when considering enrollment [40]. By preemptively clarifying risks, CRCs reduced follow-up questions and built trust. These results suggest training should continue emphasizing proactive risk communication.

The frequency analysis reinforced these findings: risk mentions in the intervention group doubled from pre- to post-training, indicating a move away from abstract reassurances toward explicit discussions of risks. The control group showed only marginal change, suggesting protocol exposure alone does not improve risk communication.

### Benefit framing: balancing uncertainty and incentives

A second major shift was increased use of participant-centered benefit framing. At Time 1, CRCs emphasized societal benefits while neglecting personal benefits, often leading to skepticism. From a UMT perspective, this failed to manage uncertainty in a personally meaningful way [24]. Participants require clear reasons for how enrollment impacts their health.

Post-training, CRCs provided balanced framing, ensuring participants understood both potential personal benefits and broader contributions to research. Balanced framing supports realistic expectations and reduces emotional discomfort associated with uncertainty. These improvements address prior concerns that trial benefits are under-communicated [40].

Frequency analysis confirmed this shift. Both groups mentioned benefits more often at Time 2, but the nature differed: the control group reduced direct benefit mentions, while the intervention group increased both direct and potential benefits. Training thus encouraged CRCs to frame benefits in ways more relevant to participants’ lived experiences.

### Participant engagement and trust-building: managing affective uncertainty

Training also improved active engagement strategies, including inviting questions and validating concerns. Such techniques are central to managing affective uncertainty – emotional distress during high-stakes decisions [41]. Affective uncertainty is best reduced through interpersonal trust-building and open dialogue [42,24].

Before training, CRCs often closed conversations by placing the burden on participants to ask questions, discouraging engagement. After training, they proactively invited questions, checked understanding, and responded empathetically rather than with dismissive reassurances. These changes reflect a shift toward relationship-building strategies essential for reducing anxiety and fostering trust [42–44].

Such transformations were absent in the control group, where communication remained largely unchanged. This contrast indicates that improvements stemmed from the training rather than familiarity with the study protocol.

### Implications for CRC training and recruitment

A persistent challenge in informed consent is making study details meaningful and understandable [44]. This study demonstrates that structured communication training enhances CRC effectiveness in recruitment. By strengthening skills in transparent risk disclosure, balanced benefit framing, and trust-building, training fosters ethical and effective participant engagement.

Theoretically, the findings extend UMT by showing that managing uncertainty in recruitment depends not only on the provision of information but also on how it is framed and conveyed. Since failed recruitment is a leading cause of trial failure [5,6], training programs should integrate transparency, proactive engagement, and message tailoring. Practically, training strengthens ethical recruitment by promoting clarity, trust, and dialogue – moving beyond compliance to participant-centered communication. CRCs often struggle with risk communication [23], but targeted training equips them to manage uncertainty about risks, procedures, and study goals. When procedures are framed as beneficial and emotional factors acknowledged, participants feel more supported. Informed consent, too often reduced to a signature, should be reimagined as a process of shared meaning-making. Addressing the scarcity of such programs [12,45], this study highlights training as a pathway to more transparent and relational recruitment.

### Limitations

This study has limitations. The small sample size limits generalizability, though the within-subject design adds analytical depth. The trial context – a low-risk blood pressure study using established medications – may have shaped CRC approaches to uncertainty, limiting applicability to more complex or high-risk trials. Finally, while SPs provided consistency, they may not capture the full variability of real-world encounters, potentially constraining how CRCs adapted strategies.

### Conclusion

Structured communication training transforms CRCs’ approaches to risk-benefit discussions and participant engagement. Grounded in UMT, the findings show that CRC communication manages uncertainty and fosters trust. Post-training, CRCs shifted from minimizing to transparently addressing risks, from generic to participant-centered benefit framing, and from passive to interactive engagement. These changes created more ethical, transparent, and participant-focused interactions. The results underscore the value of CRC training centered on uncertainty management, trust-building, and ethical engagement. Future research should examine long-term effects on recruitment, retention, and overall trial success. Informed consent in clinical trials is often hindered by participants’ limited understanding of study risks, benefits, and procedures. CRCs play a central role in this process but rarely receive structured training in communication. This study demonstrates that communication training helps CRCs engage participants more ethically and transparently, improving risk–benefit discussions, building trust, and supporting participant-centered decision-making. Enhancing CRC communication skills is a health promotion strategy that strengthens ethical recruitment, improves informed consent, and supports equitable participation in research.
